# Candies and Sweets

**Published:** 1891-04

**Authors:** 


### CANDIES AND SWEETS.

The civilized "sugar tooth" is a monstrosity, although it is a taste which is cultivated from babyhood with most American children. As to candies they are something more than sweets ; they are sweets adulterated with things which are harmful ; for it is almost impossible to get candies made from pure sugar. The "sugar" from which most of them are made grew in a cornfield—an article known to commerce as glucose. At one time we wanted some glucose very much, and had been trying vainly to get it, when some one suggested that we try a candy manufactory. We did not suppose the proprietor would own to using the stuff, but he did, and sold us all we wanted at four cents a pound. We asked him if he thought it was fit to eat, and he replied, "No; I wouldn't eat it for any thing. I make the candy for other people to eat." There is no doubt but many children are kept sick and fretful, especially with stomach and bowel troubles, from having too much sweets. Sugar causes an excessive flow of mucus, and brings on catarrh of the stomach, often while children are very young ; and it is a most obstinate disease to cure. Give the little ones oat-meal and preparations of wheat, which are half starch. In the process of digestion, this starch will be changed into sugar, and in this way they will be furnished with all the sweets their systems require, if with the grains they take plenty of fruits, with their natural, healthful sweets. If they are not taught to like candies and other artificial "goodies," they will have no appetite or craving for them.—*Good Health.*

